# Editorial: Advances in reproductive biotechnologies in carnivores

**DOI:** 10.3389/fvets.2023.1123368

**Published:** 2023-05-26

**Authors:** Monica De Los Reyes, Nucharin Songsasen, Jason Herrick

**Affiliations:** ^1^Faculty of Veterinary Sciences, University of Chile, Santiago, Chile; ^2^Center for Species Survival, Smithsonian National Zoo and Conservation Biology Institute, Front Royal, VA, United States; ^3^Omaha's Henry Doorly Zoo and Aquarium, Omaha, NE, United States

**Keywords:** carnivore, assisted reproductive techniques, theriogenology, cat, dog

The order Carnivora consists of 231 species representing 93 genera within seven families, including *Felidae, Canidae, Ursidae, Procyonidae, Mustelidae, Viverridae, and Hyaenidae*. Of the 7 families, knowledge about reproductive biology and the development of reproductive technologies have been described only in felids, canids, ursids and mustelids (1). Successful development and applications of biotechnologies to diagnose and address reproductive disorders and to preserve valuable genetics has relied on knowledge gained from studies of model species, especially the domestic dog and cat. For example, knowledge gained from the domestic cat has led to the development of assisted reproductive technologies, including sperm cryopreservation, artificial insemination and *in vitro* embryo production that leads to the production of live offspring in several wild felid species (2). In this topic, we collect four research articles that address infertility and advance reproductive technologies in domestic dogs and cats. The findings obtained from these articles will not only inform future studies in the wild cousins, but also may provide insights into the causes of some human reproductive disorders.

Non-obstructive azoospermia (NOA), the condition characterized by the lack of spermatozoa within an ejaculate, is a common cause of infertility in male dogs. Interestingly, NOA has been implicated as a major cause of infertility in the human (3). The paper by Goericke-Pesch et al. identifies histopathological changes of the testicular tissue that were characteristic of chronic, immune-mediated orchitis, including severe disruption of spermatogenesis with generalized immune cell infiltration. Despite being infertile, none of the affected animals showed any clinical signs of inflammation, which underscore the importance of developing early diagnostic tools and identifying therapeutic options for this disorder.

Benign prostate hyperplasia (BPH) is the most prevalent prostatic disease (95% in dogs ≥9 years old) in older, intact male dogs (4). This disorder severely compromises seminal quality, which in turn affects fertility. Studies have shown that ejaculates of BPH patients contain high proportion of structurally abnormal sperm with DNA fragmentation and alteration of motility kinetics, all of which can contribute to the high susceptivity to cryo-injuries. Flores et al. investigated the effects of finasteride treatment on post-thaw survival of sperm obtained from of BPH dogs. The authors demonstrated that treating BPH individuals with finasteride can improve ejaculate quality and sperm cryotolerance. The findings provide a useful approach to allow banking of sperm from genetically valuable BPH donors.

The major limitation of the utilization of cold storage of sperm samples is the gradual decrease of gamete viability and function overtime. The ability to extend the longevity of cold stored sperm will aid transportation of sperm samples across geographically isolated locations. Martínez-Barbitta and Salinas explored the value of a commercial sperm activator (SA) extender in preserving dog semen quality during a 2-week storage. The authors reported that SA extender better sustained sperm motility, normal morphology, and membrane function. While promising results were obtained from SA extender, the next logical step is to assessing fertility of sperm stored in this extender to confirm its practical potential in dog breeding.

Genome resource banks have played an important role in species conservation effort, including carnivores (1). Most sperm cryopreservation protocols employ a conventional slow-freezing method that may not be practical when conducted under field conditions. Colombo et al. investigated the impacts of two rapid freezing methods (pellet vs. straws) and extender types on the survival of cat epididymal spermatozoa. The authors demonstrated sperm cryopreserved using the pellet method (pipetting directly into liquid nitrogen) with Tris buffer + 20% egg yolk + 0.25 sucrose were able to sustain motility and structural integrity for 6 h post-thaw. Sperm cryopreserved using this method were able to fertilize cat oocytes resulting in embryonic development, and thus offer a practical approach to banking genetic materials of wild felids under field conditions.

The field of reproductive technologies includes the interactions of many disciplines, this Research Topic summarized several of these disciplines in carnivores.

## Author contributions

All authors listed have made a substantial, direct, and intellectual contribution to the work and approved it for publication.
